# Genomic optimum contribution selection and mate allocation using JuMP

**DOI:** 10.1093/bioadv/vbaf259

**Published:** 2025-10-22

**Authors:** Patrik Waldmann

**Affiliations:** Research Unit of Mathematical Sciences, University of Oulu, Oulu, FIN-90014, Finland

## Abstract

**Motivation:**

Artificial selection improves desired traits, but reduces genetic diversity within populations. Modern breeding programs aim to balance genetic gain with the maintenance of genetic variation to ensure long-term sustainability. Optimum contribution selection (OCS) is a widely adopted strategy that maximizes genetic gain while limiting the rate of inbreeding, traditionally relying on pedigree data. However, genomic relationship matrices offer a more accurate measure of genetic relatedness. A subsequent step to OCS involves mate allocation (MA) to optimize breeding plans, which often presents significant computational challenges for large datasets.

**Results:**

We developed a two-stage genomic OCS and mate allocation (GOCSMA) method implemented in JuMP/Julia. The OCS problem is formulated as a linear program with quadratic constraints and solved efficiently using the conic operator splitting method (COSMO). The subsequent MA problem, expressed as a mixed integer program, is solved with the SCIP framework’s branch-cut-and-price algorithm. Applying GOCSMA to the simulated QTLMAS2010 dataset, we observed efficient convergence for OCS, balancing genetic gain with coancestry constraints better compared to traditional top selection. The MA stage consistently achieved very low runtimes (<0.01 seconds), with integer mating constraints providing lower coancestry and higher genetic gain compared to binary constraints, indicating a more optimal mating scheme.

Hence, GOCSMA provides an efficient deterministic mathematical optimization framework for integrated genomic OCS and MA. Using advanced solvers within the flexible JuMP environment, our method offers a robust solution to balance genetic gain and diversity in large-scale breeding programs.

**Availability and implementation:**

Source code and documentation are available at https://github.com/patwa67/GOCSMA

## 1 Introduction

Artificial selection leads to a reduction in genetic diversity within a population is a well-known phenomenon in quantitative genetics. The first to establish a proper theory for this phenomenon was Johannsen who clearly made the distinction between the genotype and the phenotype. Moreover, he conducted experiments with self-fertilizing bean plants where he showed that once the genetic variation within a pure line was exhausted, further selection had no effect (Johannsen 1909). The understanding that selection leads to a reduction in additive genetic variance was a developing concept in the early 20th century, with significant contributions from Lush, Wright, and Fisher. They all recognized that the fuel for the response to selection is additive genetic variance that is consumed by the very act of selection (Lush 1937, Fisher 1958, Wright 1968). This understanding is crucial for studying the consequences of natural and artificial selection, and for designing long-term breeding programs. Today, there is a vast literature that confirms that genetic variation decreases under intense directional selection (Walsh and Lynch 2018).

Recent years have seen significant research on designing strategies that balance genetic gain and the maintenance of genetic diversity within selection programs. The main goal is to simultaneously optimize these two objectives, either by reducing the rate of inbreeding while maintaining a target level of genetic gain, or by enhancing selection response given a defined limit on inbreeding. These strategies can be categorized by their focus: (i) the criteria used for selection; (ii) the implemented mating system; and (iii) the quantity of selected individuals and their impact on the following generation (Toro and Pérez-Enciso 1990).

Lindgren *et al.* (1993) presented a method to find the optimal proportions of selected individuals within large individual families by identifying the family contributions that maximize genetic gain at a given diversity and selected proportion. They showed that the optimally selected proportion of members from a family is dependent on the average breeding value (BV) of the family, the average selected proportion, the diversity, the heritability, and the intraclass correlation for the family type. Their work highlighted the importance of moving beyond simple top selection based solely on BVs. Meuwissen (1997) further elaborated on this concept and suggested a general and comprehensive framework for optimum contribution selection (OCS) applicable across various animal breeding scenarios, explicitly formulating it as an optimization problem with the goal of maximizing genetic gain under a defined rate of inbreeding. OCS has become a widely adopted breeding strategy and serves as the basis for many subsequent developments and applications. Most OCS problems are solved with mathematical optimization (Woolliams *et al.* 2015). A variety of methods have been used for OCS, among the most popular are Lagrange multipliers (Meuwissen 1997), the interior point method with semidefinite programming formulation (Pong-Wong and Woolliams 2007) and evolutionary algorithms (Berg *et al.* 2006). However, most of the software packages for OCS are rather inflexible and the underlying algorithms may not always find the global optimum (Wellmann 2019).

A common step after OCS is to establish a scheme for mate allocations (MA). The idea is to jointly optimize contributions and MAs via optimization of a mating plan. This joint optimization mostly does not have an analytical form and is usually solved with stochastic or metaheuristic methods, such as simulated annealing and evolutionary search algorithms (Kerr *et al.* 1998, Kinghorn 2011, Akdemir and Sánchez 2016, Gorjanc and Hickey 2018, Yoshida *et al.* 2020, Niehoff *et al.* 2024). These methods are flexible in accommodating constraints and multiple objectives but normally require extensive computing resources and there is no guarantee that they reach the final global best solution. Recently, it has been shown that deterministic mathematical programming can also be successfully used for MA (Mullin and Belotti 2016, Wellmann 2019, Bérodier *et al.* 2021, Endelman 2025). Although this idea is not new (Jansen and Wilton 1985), the approach has been difficult to implement on larger scales for computational reasons, because MA is a combinatorial optimization problem that has to be reformulated as a convex problem using some form of relaxation.

Traditionally, OCS and MA have relied on pedigree-based relationship matrices. However, genomic relationship matrices G, constructed from dense single nucleotide polymorphism (SNP) marker data, provide a more accurate and precise measure of true genetic relatedness (VanRaden 2008). This means that G-matrices capture the actual proportion of the genome shared between individuals, rather than just the expected proportion based on pedigree records. Hence, genomic relationships can account for the random segregation of genes during meiosis (i.e. Mendelian sampling), which pedigree relationships cannot. Sonesson *et al.* (2012) concluded that genomic selection requires genomic control of inbreeding, i.e. genomic optimal contribution selection (GOCS). One obstacle here is that G-matrices can be calculated in several ways and there seems to be no general consensus on which one to prefer in GOCS (Meuwissen *et al.* 2020, Gautason *et al.* 2023).

The purpose of this study is to propose a two-stage genomic OCS and MA (GOCSMA) method that scales favorably to large data sets because of its efficient deterministic mathematical optimization algorithms. The OCS problem is formulated as a linear program with quadratic constraints (LPQC) and solved with the recently proposed conic operator splitting method (COSMO) (Garstka *et al.* 2021). The subsequent MA problem is expressed as a mixed integer program (MIP) that is solved with the branch-cut-and-price algorithm embedded in the constraint integer programming framework SCIP (Bolusani *et al.* 2024). Both problems are implemented and solved with the JuMP modeling language (Lubin *et al.* 2023) for mathematical optimization embedded in Julia (Bezanson *et al.* 2017). The use of GOCSMA is illustrated using the simulated QTLMAS2010 data set (Szydlowski and Paczyńska 2011).

## 2 Methods

### 2.1 Optimum contribution selection

We start by defining the OCS problem as a linear program with quadratic constraints


(1) $$\text{maximize}\;c^{T}\hat{u}$$

(2) $$\text{subject}\;\text{to}\;c^{T}Gc/2\leq \theta$$

(3) $$c^{T}s=0.5$$

(4) $$c^{T}d=0.5$$

(5) c≥0

where $c\in R^{n}$ is a 1×n vector of individual contributions, $\hat{u}$ is a vector with estimated genomic BVs, $G\in R^{n\times n}$ contains pair-wise genomic relationship coefficients of the *n* available individuals, θ is the maximum allowed coancestry in the next generation and the lowest possible contribution is 0. The constraint on gender contribution, expressed here as the vectors s and d in constraints (3) and (4), can be changed if there is a practical reason for that.

### 2.2 Conic operator splitting method

COSMO is a solver for convex conic optimization problems that uses the alternating direction method of multipliers (ADMM) to solve problems that can be formulated in a standard form (Garstka *et al.* 2021). To use COSMO for the OCS problem, we must re-express our specific formulation (1)–(5) into the following generalized form


(6) $$\text{minimize}\;\frac{1}{2}x^{T}Dx+q^{T}x$$

(7) subject to Ax+y=b

(8) y∈X

The COSMO solver interprets the variables from our OCS problem as follows

- The decision vector x is our vector of individual contributions, c.
- The objective function of the OCS problem is to maximize $c^{T}\hat{u}$. To fit the standard minimization form, we convert this to minimizing $-c^{T}\hat{u}$. The matrix D for the quadratic part is the genomic relationship matrix, G, scaled by 1/2 (from the coancestry term). The vector q for the linear part is the vector of estimated genomic BVs, $-\hat{u}$.
- The linear equality constraints defined by Ax+y=b represent our gender contribution rules ($c^{T}s=0.5$ and $c^{T}d=0.5$).
- The slack variable y and the convex set X are used by the solver to handle our remaining constraints, which include the quadratic coancestry constraint ($c^{T}Gc/2\leq \theta$) and the non-negativity constraint (c≥0).

This reformulation allows the COSMO solver to efficiently find the optimal contribution vector c that balances genetic gain and inbreeding, as defined by the OCS problem.

The ADMM is an iterative optimization algorithm that solves a complex problem by breaking it down into a sequence of smaller, easier-to-solve subproblems (Boyd *et al.* 2010, Beck 2017). For the OCS problem, COSMO reformulates the optimization to fit the standard ADMM form as


(9) $$\underset{c,z}{\min}f(c)+g(z)\;\text{subject}\;\text{to}\;\mathrm{Ac}+\mathrm{Bz}=0$$

where f(c) represents the objective function and the quadratic coancestry term, g(z) is an indicator function that is zero when the linear constraints (gender contributions) and non-negativity bounds are met, and infinity otherwise. The linear constraint Ac+Bz=0 enforces that the split variables c and z are in agreement throughout the process. In ADMM, we first denote a dual variable as λ. The algorithm then proceeds through three main steps in a repeating loop as

1. The c-update: The algorithm updates the contribution vector c by minimizing a subproblem that includes the original objective function and a penalty term to encourage agreement with the auxiliary variable z
 
  (10) $$c^{k+1}=\underset{c}{\arg\min}\left(\frac{1}{2}c^{T}Gc-{\left(\hat{u}\right)}^{T}c+\frac{\gamma }{2}{\left\|c-z^{k}+\lambda^{k}\right\|}_{2}^{2}\right).$$
  This step is often a quadratic subproblem that can be solved very efficiently.
2. The z-update: The auxiliary variable z is updated by projecting onto the feasible set of the problem’s constraints
  (11) $$z^{k+1}=\underset{z\in X}{\arg\min}\left(\frac{\gamma }{2}{\left\|c^{k+1}-z+\lambda^{k}\right\|}_{2}^{2}\right).$$
  This step ensures that the updated variables adhere to the specified cones and linear equalities.
3. The dual-update: Then the dual variable λ is updated as
  (12) $$\lambda^{k+1}=\lambda^{k}+(c^{k+1}-z^{k+1}).$$

This variable acts as a Lagrange multiplier, penalizing violations of the consensus constraint Ac+Bz=0 and driving the two sets of variables toward a single solution.

The iterative process continues until the difference between successive updates is below a predefined tolerance, indicating that the algorithm has converged to an optimal solution. The condition for convergence is a combination of an absolute and a relative tolerance, which accounts for the scale of the problem. The primal residual, denoted as $r^{k}$, measures the distance between the split variables c and z at iteration *k*. It is a measure of how well the consensus constraint is being satisfied and defined as


(13) $$r^{k}=c^{k}-z^{k}$$

The primal residual must be below a tolerance that scales with the magnitude of the variables. The condition for primal convergence is


(14) $$∥r^{k}{∥}_{2}\leq \epsilon_{\text{abs}}+\epsilon_{\;\text{rel}}\max(∥c^{k}{∥}_{2},∥z^{k}{∥}_{2})$$

This means the residual must be small in an absolute sense, but also small relative to the norms of the primal variables. The dual residual, denoted as $s^{k}$, is related to the change in the auxiliary variable from one iteration to the next, scaled by the penalty parameter γ. It represents the dual optimality condition and is defined as


(15) $$s^{k}=\gamma (z^{k}-z^{k-1})$$

The dual residual must be below a tolerance that scales with the magnitude of the dual variable λ. The condition for dual convergence is


(16) $$∥s^{k}{∥}_{2}\leq \epsilon_{\text{abs}}+\epsilon_{\;\text{rel}}∥\lambda^{k}{∥}_{2}$$

The ADMM algorithm in COSMO will stop when both the primal and dual termination criteria are satisfied. The default values for both $\epsilon_{\text{abs}}$ and $\epsilon_{\text{rel}}$ are $1\times 10^{-5}$.

The constant γ is a penalty parameter that helps control the convergence rate. The default value for γ in the COSMO solver is typically 0.1. However, it is important to note that COSMO uses an adaptive γ scheme by default. This means the initial value of 0.1 is often just a starting point, and the solver will dynamically adjust γ during the optimization process to improve convergence. In practice, COSMO will not set the values of the contribution vector c to exactly zero, so we set values below a small threshold ρ=0.0001 to zero, i.e. [c<ρ]=0.

### 2.3 Mate allocation

We first extract individuals with nonzero contributions into one new MA vector for females $c_{d}=\{[c>0]|[d=1]\}$ of size $1\times n_{d}$ and one vector for males $c_{s}=\{[c>0]|s=1\}$ of size $1\times n_{s}$. Then we use these individuals to create a new genomic relationship matrix $H\subseteq [G|c_{d},c_{s}]$ of size $n_{d}\times n_{s}$ that will contain pairwise genomic relationship coefficients of the selected individuals. There are several possibilities to create MA schemes and we will start with a framework where matings are binary and proportional to the contributions of both sexes, but with a constraint on the number of matings on the females. This results in a mixed binary linear program (BLP) with linear constraints following


(17) $$\text{minimize}\;c_{d}^{T}(H⊙M)c_{s}$$

(18) $$\text{subject}\;\text{to}\;\sum_{j}M_{j}\leq \kappa_{d}$$

(19) $$\sum_{i}M_{i}\geq \kappa_{s}$$

(20) $$M_{i,j}\in \{0,1\}$$

where ⊙ denotes the Hadamard product, M is a mating matrix (indexed by *i* and *j*) of size $n_{d}\times n_{s}$, $\kappa_{d}$ is the maximum number of matings for each female and $\kappa_{s}$ is the minimum number of matings for each male. We have chosen to set $\kappa_{s}=0$ which will prune male individuals with very small contributions. One could consider using $\kappa_{s}=1$, but this may lead to numerical issues and convergence problems because some contributions are very small. The binary mating constraint in (20) can easily be changed to integer mating constraints using $M_{i,j}\in \{0,1,\ldots ,\sigma_{d}\}$ and the model becomes an integer linear program (ILP). Note that this problem is not quadratic because $c_{d}$ and $c_{s}$ are constants and therefore are treated as linear constraints. This can be shown by first defining a new matrix Q=H⊙M. Since $c_{d}$ and $c_{s}$ are known constant vectors, their elements are fixed numerical values and the expression $c_{d}^{T}Qc_{s}$ can be expanded as a sum


(21) $$\sum_{i}\sum_{j}c_{d,i}Q_{i,j}c_{s,j},$$

and now substituting $Q_{i,j}=H_{i,j}\cdot M_{i,j}$ in (12) we can rearrange into


(22) $$\sum_{i}\sum_{j}\left(c_{d,i}H_{i,j}c_{s,j}\right)M_{i,j}.$$

Since $c_{d}$, $c_{s}$, and H are constants, their product $C_{i,j}=c_{d,i}H_{i,j}c_{s,j}$ is also a constant and the problem reduces to


(23) $$\sum_{i}\sum_{j}C_{i,j}M_{i,j}.$$

This objective function is a linear combination of the binary variables in M because there are no quadratic $M_{i,j}^{2}$ or interaction terms $M_{i,j}M_{k,l}$. While the objective function is linear and therefore a convex function, a BLP is not a convex optimization problem. This is because the feasible region of a BLP is a set of isolated points (the vertices of a hypercube), which is not a convex set. Convex optimization requires both the objective function and the feasible region to be convex. The difficulty in solving BLPs arises from the discrete nature of the decision space, which is precisely what makes the feasible region non-convex and the problem NP-hard. NP-hard problems are particularly challenging for optimization because finding the exact optimal solution for large instances of these problems is computationally infeasible. Although the general BLP problem can be transformed into a convex quadratic programming problem (Munapo 2016), it is more common to resort to convex relaxation of the binary constraints and use specialized algorithms such as interior point or branch-price-and-cut (Petris *et al.* 2025).

### 2.4 Solving constraint integer programs

SCIP (Solving Constraint Integer Programs) is a powerful and versatile optimization solver and framework that can handle mixed integer linear programs, mixed integer quadratic programs, and general mixed integer non-linear programs with a large range of constraints (Bolusani *et al.* 2024). While SCIP can be configured in many ways, its core approach for solving MIPs, which includes BLPs, is branch-and-cut. SCIP starts by solving the LP relaxation of the original BLP. This means temporarily ignoring the binary (or integer) constraints and allowing the decision variables to take any real value between their bounds $0\leq M_{i,j}\leq 1$. If the LP relaxation solution is not integer-feasible (i.e. some $M_{i,j}$ are fractional), SCIP selects a fractional variable $M_{i,j}^{*}$ and creates two subproblems by branching. For a binary variable, one branch fixes $M_{i,j}^{*}=0$ and the other fixes $M_{i,j}^{*}=1$. This process continues recursively, forming a search tree. At each node of the tree, an LP relaxation is solved. The optimal objective value of this LP relaxation provides a lower bound for minimization problems on the optimal solution of that subproblem and all problems in its subtree. A branch is pruned if a subproblem’s LP relaxation is infeasible. If a subproblem’s LP relaxation solution is integer-feasible, it is a candidate for the overall optimal solution. Its objective value then provides an upper bound on the global optimum. If a node’s lower bound is greater than or equal to the current best known upper bound, that branch can be pruned because it cannot lead to a better integer solution.

SCIP also employs various cut generators (separators) when an LP relaxation is solved and yields a fractional solution. These generators identify valid inequalities (cutting planes) that are violated by the current fractional LP solution but are valid for the integer feasible region. Adding these cuts to the LP relaxation tightens the feasible region of the relaxation, leading to a stronger (higher) lower bound, which can significantly reduce the size of the branch-and-bound tree. SCIP has a wide array of built-in cut types [e.g. Gomory cuts, knapsack cuts, flow cover cuts, and mixed-integer rounding (MIR) cuts]. In addition, SCIP applies powerful presolving techniques before the main branch-and-cut algorithm begins. These transformations tighten variable bounds, reduce the number of variables and constraints, and identify fixed variables which can drastically reduce the size and complexity of the problem, leading to faster solution times. Finally, throughout the branch-and-cut process, SCIP employs a large suite of primal heuristics. These algorithms are designed to quickly find good feasible (integer) solutions, even if they are not necessarily optimal. For further details of the SCIP framework see (Bolusani *et al.* 2024).

### 2.5 Implementation in JuMP/Julia

JuMP (Julia for Mathematical Programming) is a powerful and popular modeling language embedded in Julia that allows users to formulate and solve a wide variety of mathematical optimization problems (Lubin *et al.* 2023). It is not a solver itself, but rather an interface that allows you to define your problem in a high-level easy-read code and then pass it to various specialized optimization solvers (both commercial and open-source). JuMP can be used for linear programming, mixed-integer programming, quadratic programming, conic programming, and nonlinear programming. Its syntax closely resembles mathematical expressions, making it intuitive for those familiar with optimization theory. It uses a generic interface (MathOptInterface) that makes it easy to switch between different open-source and commercial solvers without changing the problem formulation. Being written purely in Julia, it seamlessly integrates into larger Julia workflows, allowing optimization problems to be solved as part of simulations, web applications, or decomposition algorithms. GOSCMA is implemented in JuMP and the code is available at: https://github.com/patwa67/GOCSMA

In order to further evaluate the statistical properties of the binary and integer versions of GOCSMA, we calculated the future coancestry as


(24) $$\theta_{f}=\frac{1}{N_{\mathrm{matings}}}\sum_{i,j}M_{i,j}\times G_{i,j},$$

where $M_{i,j}$ is the number of matings between dam *i* and sire *j*, $G_{i,j}$ is the coancestry between dam *i* and sire *j* and $N_{\mathrm{matings}}$ is the total number of matings. Moreover, the future genetic gain was obtained as


(25) $$\Delta \hat{u}=E({\hat{u}}_{\mathrm{sel}})-E({\hat{u}}_{\mathrm{pop}}),$$

where $E({\hat{u}}_{\mathrm{pop}})$ is the average BV of the entire breeding population and $E({\hat{u}}_{\mathrm{sel}})$ the weighted average of the parents’ BVs where the weights are the number of matings. The corresponding estimates of $\theta_{f}$ and $\Delta \hat{u}$ were obtained for genomic OCS followed by random mating between selected sires and dams (GOCSRM) and for ordinary truncation selection followed by random mating (TSRM) with the same number of total matings $\left(N_{\mathrm{matings}}\right)$ as for GOCSMA. The two random mating schemes were repeated 100× and averaged.

### 2.6 Simulated QTLMAS2010 data

The simulated data was produced for the 14th European workshop on QTL mapping and marker-assisted selection (QTL-MAS) (Szydlowski and Paczyńska 2011). It consists of a five-generation pedigree of 3226 individuals, originating from 20 founders (5 males, 15 females). The pedigree structure assumed that each female mated once and produced ∼30 offspring. Parents for subsequent generations were primarily selected from the current generation, with a small probability of selection from older generations, resulting in nearly discrete generations. Five autosomal chromosomes were simulated, each about 100M bp long. 9345 SNPs out of 10 031 had MAF >0.05 and were retained for further analysis. Two phenotypes were simulated: a quantitative trait (QT) and a binary trait (BT), both influenced by 37 quantitative trait loci (QTLs). These QTLs comprised 9 controlled genes and 28 random genes, all pre-selected from simulated SNPs. In this study, we use only the QT for which the controlled genes were chosen based on high polymorphism and strong linkage disequilibrium (LD) with markers. These included two pairs of epistatic genes, three maternally imprinted genes, and two additive major genes. Random QT genes were sampled from simulated SNPs (excluding chromosome 5), with their additive effects drawn from a normal distribution. Residuals were uncorrelated and drawn from normal distributions with variances of 51.76. The narrow-sense heritability for the QT was 0.52 for males and 0.39 for females. The BVs were obtained as the sum of 30 additive QTLs, haplotype effects of epistatic QTLs, and imprinted QTLs (for males only). The genomic relationship matrix was calculated following VanRaden method 1 as $G=ZZ^{T}/(2\sum_{j}p_{j}(1-p_{j}))$, where Z=N−P and N is the genotypic matrix for genotyped individuals. The rows of N are genotypes with values 2 or 0 for homozygotes and 1 for heterozygotes. The columns of N correspond to the marker loci. P is a matrix in which all elements in the *j*th column are $2p_{j}$with $p_{j}$ being the frequency of the allele that is counted in N locus *j* (VanRaden 2008).

## 3 Results

### 3.1 Optimum contribution selection

The number of iterations to convergence for COSMO was considerably larger with θ=0.01 (3980) than with θ=0.001 (490). The iteration difference is relatively well reflected in the runtime where θ=0.01 is 2.9× slower. We can also note that the objective value $c^{T}\hat{u}$ is higher for θ=0.001 with −26.43 than for θ=0.01 with −32.33. Both the number of selected dams (females) and number of selected sires (males) were higher for the stricter coancestry level at θ=0.001. Note also that the number of selected dams was higher than the number of selected sires at both θ levels. Moreover, the mean of the coancestry of selected individuals in matrix H was as expected higher for θ=0.01 than for θ=0.001. In addition, the mean of the optimum contributions in c and mean of the BVs were also higher for θ=0.01 than for θ=0.001. Table 1 summarizes the performance of the COSMO solver in the OCS optimization with θ=0.01 and θ=0.001.

**Table 1.** Statistics from the OCS optimization using the COSMO solver for two different levels of coancestry constraints θ.^a^

	θ=0.01	θ=0.001
Iterations to convergence	3980	490
Optimum objective value	−32.33	−26.43
Runtime	127 s	44 s
Number of selected dams	50	155
Number of selected sires	35	142
H mean	0.000516	−0.00650
c mean	0.0118	0.00336
$\hat{u}$ mean	29.54	24.78

a **H** is the coancestry matrix, **c** is the vector with contributions, and $\hat{u}$ is the vector with breeding values of the selected individuals.

**Figure 1.**
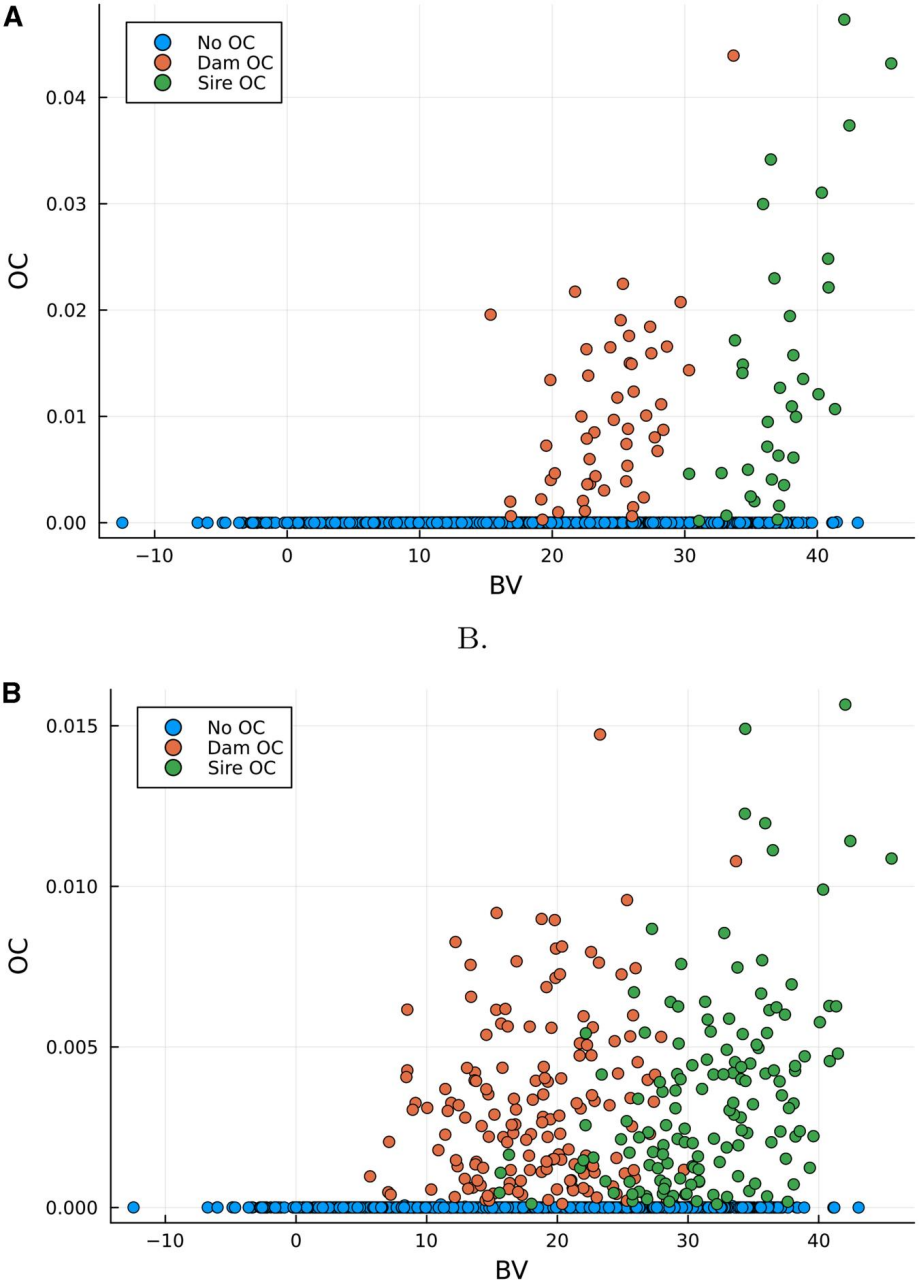
Optimum contribution values (OC) vs. breeding values (BV) of selected females (dam), males (sire) and not selected individuals of the simulated QTLMAS2010 data. (A) Coancestry constraint θ=0.01, (B) Coancestry constraint θ=0.001.

Figure 1 provides plots of the optimum contribution values (OC) against the BVs of selected females (dam), males (sire), and not selected individuals of the simulated QTLMAS2010 data.

### 3.2 Mate allocation

The runtime of SCIP was <0.01 seconds for all four (i.e. two binary and two integer) MA evaluations. The optimum objective values from $c_{d}^{T}(H⊙M)c_{s}$ were lower for the integer MA constraints (−0.0062 for θ=0.01 and −0.0024 for θ=0.001) compared with the binary MA constraints (−0.0047 for θ=0.01 and −0.0019 for θ=0.001). Hence, the integer MA constraints provide a better modeling alternative, since the objective was to minimize the coancestry among the selected individuals. The mating schemes are obtained from the matrix M and can be saved to text files (not presented here due to space limitations).

**Figure 2.**
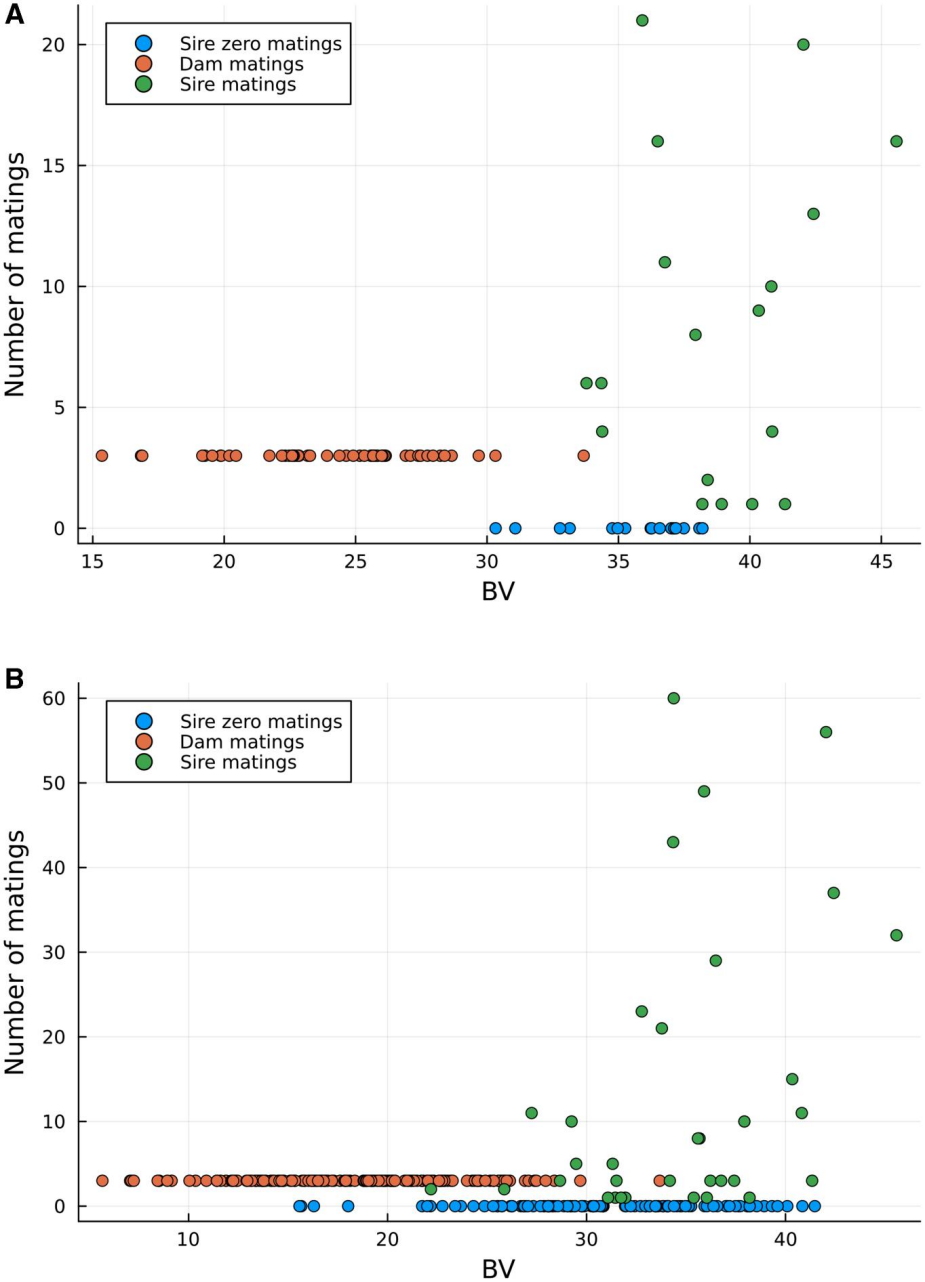
Number of mating allocations (MA) vs. breeding values (BV) of the OC selected females (dam) and males (sire) of the simulated QTLMAS2010 data when the model is restricted to binary matings. (A) Coancestry constraint θ=0.01, (B) Coancestry constraint θ=0.001.

**Figure 3.**
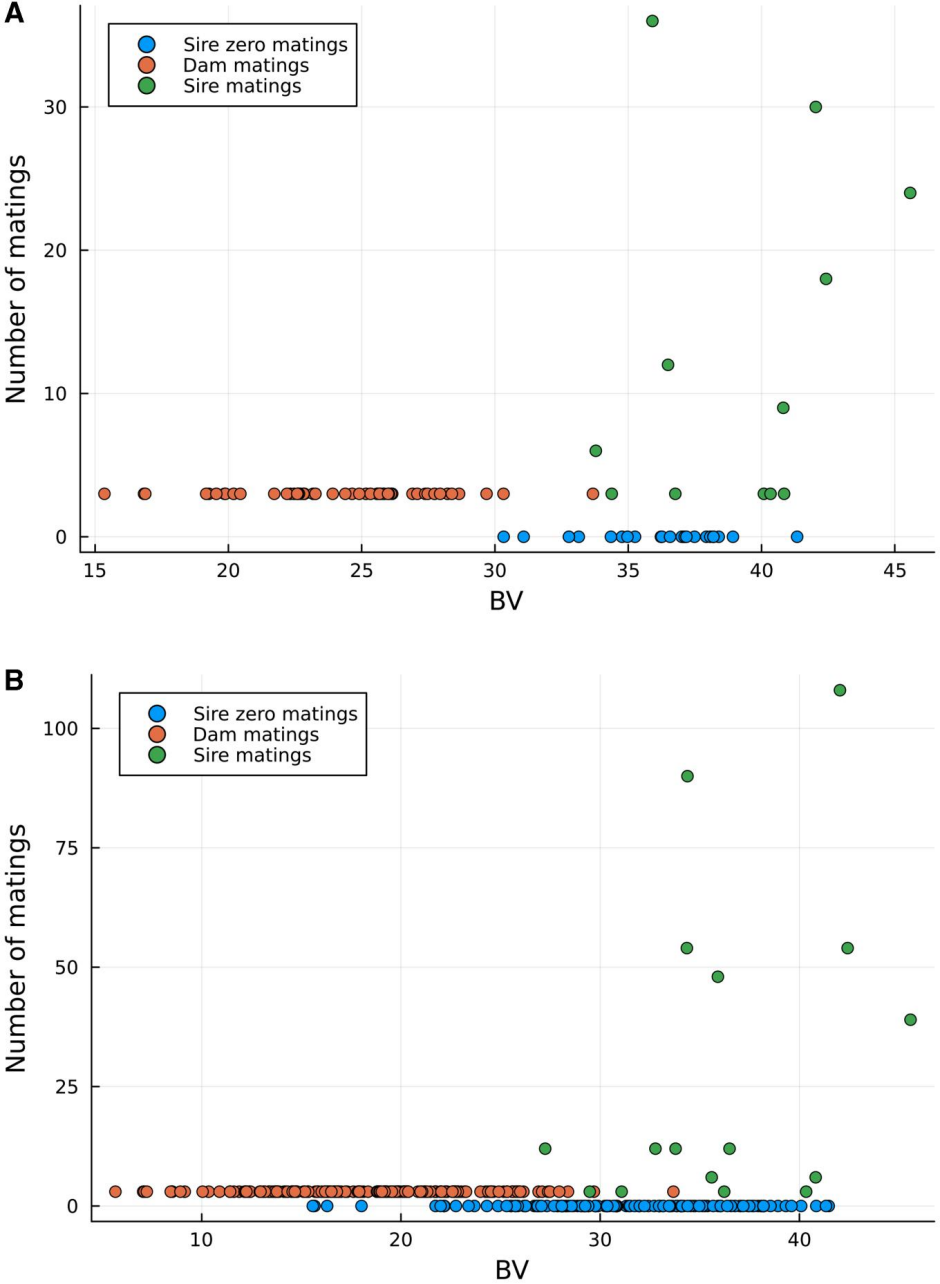
Number of mating allocations (MA) vs. breeding values (BV) of the OC selected females (dam), males (sire) of the simulated QTLMAS2010 data when the model is restricted to integer matings. (A) Coancestry constraint θ=0.01, (B) Coancestry constraint θ=0.001.

Figure 2 provides plots of the number of MA against the BVs of selected females (dam), males (sire) and not selected individuals of the simulated QTLMAS2010 data when the model is restricted to binary matings and Fig. 3 provides plots of the same parameters when the model is restricted to integer matings. We can see that the number of sires contributing to matings is higher for the binary constraints (18 for θ=0.01 and 34 for θ=0.001) than for the integer constraints (12 for θ=0.01 and 16 for θ=0.001). Moreover, the number of matings for the dams is pushed to the maximum value of three for all dams no matter which MA evaluation we run.

As expected, the future $\theta_{f}$ was in general higher for threshold θ=0.01 than for threshold θ=0.0.1. Moreover, the highest values of $\theta_{f}$ were obtained with TSRM with 0.060 for θ=0.01 and 0.029 for θ=0.001, respectively. The lowest estimates of $\theta_{f}$ were found with integer GOCSMA with −0.122 for θ=0.01 and −0.130 for θ=0.001. Binary GOCSMA and GOCSRM produced estimates between TSRM and integer GOCSMA. The largest increase in genetic gain $\Delta \hat{u}$ was achieved with TSRM with 14.89 for θ=0.01 and 11.85 for θ=0.001. The smallest improvement in $\Delta \hat{u}$ was obtained with GOCSRM which yielded 12.71 for θ=0.01 and 7.08 for θ=0.001. However, it is interesting to note that integer GOCSMA produced $\Delta \hat{u}$ estimates (14.10 for θ=0.01 and 10.30 for θ=0.001, respectively) that were quite close to TSRM. Table 2 summarizes the result of the $\theta_{f}$ and $\Delta \hat{u}$ evaluations.

**Table 2.** Estimates of the comparison of future coancestry ($\theta_{f}$) and genetic gain ($\Delta \hat{u}$) for two different levels of coancestry constraints θ.^a^

	θ=0.01	θ=0.001
$\theta_{f}$		
TSRM	0.060	0.029
GOCSRM	0.0010	−0.0062
GOCSMA Bin	−0.106	−0.118
GOCSMA Int	−0.122	−0.130
$\Delta \hat{u}$		
TSRM	14.89	11.85
GOCSRM	12.71	7.08
GOCSMA Bin	13.73	9.72
GOCSMA Int	14.10	10.30

a TSRM is truncation selection followed by random mating, GOCSRM is genomic optimum contribution selection followed by random mating, GOCSMA Bin is genomic optimum contribution selection followed by binary mating allocation, and GOCSMA Int is genomic optimum contribution selection followed by integer mating allocation.

## 4 Discussion

This study introduces GOCSMA, a two-stage genomic OCS (GOCS) and MA method implemented in JuMP, and demonstrates its performance on the simulated QTLMAS2010 dataset. The findings underscore the efficacy and computational efficiency of using deterministic mathematical optimization for the integrated management of genetic gain and diversity in breeding programs.

### 4.1 Interpretation of GOCS outcomes

The GOCS stage, solved using the COSMO solver, clearly demonstrated the expected trade-off between maximizing genetic gain and controlling coancestry. As the coancestry constraint (θ) was tightened, the optimal objective value $c^{T}\hat{u}$ decreased, confirming that achieving lower inbreeding rates requires a compromise in immediate genetic improvement. This outcome aligns with the fundamental principles of optimal contribution selection, where strict diversity management requires a broader parental contribution, consequently diluting the impact of individuals with the highest genetic merit (Meuwissen 1997, Woolliams *et al.* 2015).

A particularly noteworthy finding was COSMO’s computational efficiency. Despite the significantly higher number of iterations required for convergence under the stricter coancestry constraint (θ=0.001), the increase in total runtime was comparatively minor. This robust performance suggests that COSMO’s underlying ADMM algorithm, with its efficient handling of conic constraints, scales effectively with increased problem complexity, making it suitable for large-scale genomic datasets where computational bottlenecks are common (Garstka *et al.* 2021).

The shift to a stricter coancestry constraint also predictably led to the selection of a larger number of individuals, especially females. This outcome reflects the mechanism by which GOCS manages inbreeding. By spreading contributions across a wider pool of parents, the average coancestry within the selected group is effectively reduced. The observed consistent imbalance in selected sexes, with more dams than sires, mirrors typical breeding population structures and highlights the method’s adaptability to practical demographic constraints. The higher mean coancestry within the selected individuals under the looser constraint confirmed the direct impact of the imposed coancestry limit.

The visualizations in Fig. 1 further illustrate these dynamics. Under more relaxed θ, contributions were concentrated among individuals with very high BVs, indicating a focus on maximizing short-term gain. Conversely, the tighter θ distributed contributions more broadly among individuals with a wider range of BVs, including some with lower genetic merit.

### 4.2 Efficacy of deterministic MA

The MA stage which was optimized with SCIP, achieved remarkably fast runtimes. This exceptional computational speed and fast convergence represents a significant practical advantage over traditional stochastic or metaheuristic methods often employed for MA (Kerr *et al.* 1998, Kinghorn 2011). It is well-known from the optimization literature that deterministic methods in general show excellent results when the search space is convex and continuous. They search all space of feasible solutions and guarantee global optimality. On the other hand, for complex non-convex problems, they can be sensitive to initial parameter values and may get stuck in local optima. The stochastic methods rely on randomness and have no way of knowing when and if they have found the global optimum, they simply cannot guarantee they will find it Azevedo *et al.* (2024). Another major drawback of evolutionary inspired stochastic algorithms is that they may suffer from premature convergence if the proposal population becomes too uniform. Hence, tuning of the algorithm may turn out to be a difficult task and add considerably to computation time (Koop *et al.* 2024). SCIP’s efficient branch-and-cut algorithm, along with its presolving and cut generation capabilities, proved to be highly effective in solving this mixed integer programming problem (Bolusani *et al.* 2024). This demonstrates the feasibility of deterministic mathematical programming for MA problems on a scale previously challenging.

The comparison between binary and integer mating constraints highlighted that the integer mating scheme was superior in minimizing overall coancestry among the selected individuals. This indicates that allowing for multiple matings per individual provides greater flexibility for the optimization algorithm to identify mating pairs that collectively reduce overall relatedness, a critical factor in inbreeding management. This flexibility allows for the more strategic and efficient utilization of genetically valuable individuals.

The contrasting number of sires used under each constraint further illuminated this trade-off. Binary mating constraints demanded a larger number of contributing sires because each could only be assigned one mating. In contrast, integer mating constraints enabled the strategic reuse of fewer high-BV sires for multiple matings. Although using more sires can potentially distribute genetic risk, the ability to repeatedly use elite individuals under integer constraints can lead to a more optimized overall coancestry, even if it concentrates contributions. The consistent maximal utilization of dams’ mating capacity across all MA evaluations suggests this was a key constraint influencing mating distribution among females.

Figures 2 and 3 graphically confirm these operational differences. The integer mating scheme (Fig. 3) showed a concentrated use of high-BV males, demonstrating the optimization’s ability to achieve lower coancestry by leveraging these individuals multiple times. In contrast, the binary scheme (Fig. 2) showed matings distributed across a broader range of males, providing a different balance between genetic contribution and diversity. These insights are crucial for breeders in deciding on practical mating system implementations based on their specific goals for genetic gain versus diversity distribution.

As a final note, we can clearly see that GOCSMA with integer mating provides the lowest future coancestry level no matter which coancestry constraint we set, while it at the same time produces a future genetic gain that is close to the level obtained with standard truncation selection. The improved balance between future genetic gain and coancestry is striking when we compare GOCSMA with GOCSRM.

## 5 Conclusion

The GOCSMA methodology, with its two-stage deterministic optimization, offers a robust, flexible, and computationally efficient framework for modern genomic breeding programs. The choice of JuMP as the modeling language ensures transparency and adaptability, allowing breeders to easily tailor objectives and constraints to specific breeding goals, a significant advantage over less flexible proprietary software. The reliance on genomic relationship matrices is fundamental as they provide a more accurate measure of true genetic relatedness by accounting for Mendelian sampling that gives precision beyond pedigree-based approaches.

Although this study successfully demonstrated the utility of GOCSMA, there are several promising avenues for future research. Given the ongoing discussions in the literature (Meuwissen *et al.* 2020, Gautason *et al.* 2023), exploring the impact of different G-matrix constructions within the GOCS framework would be valuable. Additionally, extending GOCSMA to incorporate more complex multiobjective optimization, such as multiple economically important traits or specific trait-linked genetic markers, would broaden its practical applicability. It would also be interesting to evaluate the long-term effect of the combined OCS and MA strategies over several generations of selection. Finally, validating the impact of GOCSMA on genetic diversity and gain in real-world breeding populations would provide critical empirical evidence of its sustained utility and robustness. The efficient computational performance achieved in this study lays the groundwork for such comprehensive future investigations.

## Data Availability

The code for the GRVSNN models is available at: https://github.com/patwa67/GOSCMA. The original QTLMAS2010 data is available at: https://jay.up.poznan.pl/qtlmas2010/dataset.html

## References

[vbaf259-B1] Akdemir D , Sánchez JI. Efficient breeding by genomic mating. Front Genet 2016;7:210. 27965707 10.3389/fgene.2016.00210 PMC5126051

[vbaf259-B2] Azevedo BF , Rocha AMAC, Pereira AI. Hybrid approaches to optimization and machine learning methods: a systematic literature review. Mach Learn 2024;113:4055–97.

[vbaf259-B3] Beck A. First-Order Methods in Optimization. Philadelphia, PA: Society for Industrial and Applied Mathematics, 2017.

[vbaf259-B4] Berg P , Nielsen J, Kargo Sørensen M. EVA: realized and predicted optimal genetic contributions. In: Book of Abstracts. Belo Horizonte, Brazil: WCGALP, 2006, 246.

[vbaf259-B5] Bezanson J , Edelman A, Karpinski S et al Julia: a fresh approach to numerical computing. SIAM Rev 2017;59:65–98.

[vbaf259-B6] Bolusani S , Besançon M, Bestuzheva K et al The Scip Optimization Suite 9.0. 2024. https://optimization-online.org/2024/02/the-scip-optimization-suite-9-0/

[vbaf259-B7] Boyd S , Parikh N, Chu E et al Distributed optimization and statistical learning via the alternating direction method of multipliers. FNT Mach Learn 2010;3:1–122.

[vbaf259-B8] Bérodier M , Berg P, Meuwissen T et al Improved dairy cattle mating plans at herd level using genomic information. Animal 2021;15:100016. 33516018 10.1016/j.animal.2020.100016

[vbaf259-B9] Endelman J. B. Genomic prediction of heterosis, inbreeding control, and mate allocation in outbred diploid and tetraploid populations. Genetics 2025:229:iyae193. 39552210 10.1093/genetics/iyae193

[vbaf259-B10] Fisher RA. The Genetical Theory of Natural Selection. 2nd edn. New York, NY: Dover Publications, 1958.

[vbaf259-B11] Garstka M, Cannon M, Goulart P. COSMO: a conic operator splitting method for convex conic problems. *J Optim Theory Appl* 2021;**190**:779–810. https://doi.org/10.1007/s10957-021-01896-x

[vbaf259-B12] Gautason E , Sahana G, Guldbrandtsen B et al Impact of kinship matrices on genetic gain and inbreeding with optimum contribution selection in a genomic dairy cattle breeding program. Genet Sel Evol 2023;55:48. 37460999 10.1186/s12711-023-00826-x PMC10351146

[vbaf259-B13] Gorjanc G , Hickey JM. AlphaMate: a program for optimizing selection, maintenance of diversity and mate allocation in breeding programs. Bioinformatics 2018;34:3408–11. 29722792 10.1093/bioinformatics/bty375 PMC6157072

[vbaf259-B14] Jansen GB , Wilton JW. Selecting mating pairs with linear programming techniques. J Dairy Sci 1985;68:1302–5. 3842871 10.3168/jds.S0022-0302(85)80961-9

[vbaf259-B15] Johannsen W. Elemente der exakten Erblichkeitslehre. Jena, Germany: Gustav Fischer, 1909.

[vbaf259-B16] Kerr RJ , Goddard ME, Jarvis SF. Maximising genetic response in tree breeding with constraints on group coancestry. Silvae Genet 1998;47:165–73.

[vbaf259-B17] Kinghorn BP. An algorithm for efficient constrained mate selection. Genet Sel Evol 2011;43:4. 21251244 10.1186/1297-9686-43-4 PMC3037843

[vbaf259-B18] Koop L , do Valle Ramos NM, Bonilla-Petriciolet A et al A review of stochastic optimization algorithms applied in food engineering. Int J Chem Eng 2024;2024:636305.

[vbaf259-B19] Lindgren D , Wei RP, Bondesson FL. Optimal selection from families. Heredity (Edinb) 1993;70:619–21.

[vbaf259-B20] Lubin M , Dowson O, Garcia JD et al JuMP 1.0: recent improvements to a modeling language for mathematical optimization. Math Prog Comp 2023;15:581–9.

[vbaf259-B21] Lush JL. Animal Breeding Plans. Ames, IA: Iowa State Press, 1937.

[vbaf259-B22] Meuwissen THE. Maximizing the response of selection with a predefined rate of inbreeding. J Anim Sci 1997;75:934–40. 9110204 10.2527/1997.754934x

[vbaf259-B23] Meuwissen THE , Sonesson AK, Gebregiwergis G et al Management of genetic diversity in the era of genomics. Front Genet 2020;11:880. 32903415 10.3389/fgene.2020.00880 PMC7438563

[vbaf259-B24] Mullin TJ , Belotti P. Using branch-and-bound algorithms to optimize selection of a fixed-size breeding population under a relatedness constraint. Tree Genet Genomes 2016;12:4.

[vbaf259-B25] Munapo E. Solving the binary linear programming model in polynomial time. AJOR 2016;06:1–7.

[vbaf259-B26] Niehoff TAM , ten Napel J, Bijma P et al Improving selection decisions with mating information by accounting for mendelian sampling variances looking two generations ahead. Genet Sel Evol 2024;56:41. 38773363 10.1186/s12711-024-00899-2 PMC11107025

[vbaf259-B27] Petris M , Archetti C, Cattaruzza D et al A tutorial on branch-price-and-cut algorithms. 4OR-Q J Oper Res 2025;23:1–52.

[vbaf259-B28] Pong-Wong R , Woolliams JA. Optimisation of contribution of candidate parents to maximise genetic gain and restricting inbreeding using semidefinite programming. Genet Sel Evol 2007;39:3–25. 17212945 10.1186/1297-9686-39-1-3 PMC2739432

[vbaf259-B29] Sonesson AK , Woolliams JAW, Meuwissen THE. Genomic selection requires genomic control of inbreeding. Genet Sel Evol 2012;44:27. 22898324 10.1186/1297-9686-44-27 PMC3522025

[vbaf259-B30] Szydlowski M. , Paczyńska P. QTLMAS 2010: simulated dataset. BMC Proc 2011;5:S3. 10.1186/1753-6561-5-S3-S3 PMC3103202 21624173

[vbaf259-B31] Toro M , Pérez-Enciso M. Optimization of selection response under restricted inbreeding. Genet Sel Evol 1990;22:93.

[vbaf259-B32] VanRaden PM. Efficient methods to compute genomic predictions. J Dairy Sci 2008;91:4414–23. 18946147 10.3168/jds.2007-0980

[vbaf259-B33] Walsh B , Lynch M. Evolution and Selection of Quantitative Traits. Oxford, UK: Oxford University Press, 2018.

[vbaf259-B34] Wellmann R. Optimum contribution selection for animal breeding and conservation: the R package optiSel. BMC Bioinformatics 2019;20:25. 30642239 10.1186/s12859-018-2450-5 PMC6332575

[vbaf259-B35] Woolliams JA , Berg P, Dagnachew BS et al Genetic contributions and their optimization. J Anim Breed Genet 2015;132:89–99. 25823835 10.1111/jbg.12148

[vbaf259-B36] Wright S. Evolution and the Genetics of Populations. Vol. 1. Genetic and Biometric Foundations. Chicago, IL: University of Chicago Press, 1968.

[vbaf259-B37] Yoshida GM , Yáñez JM, de Queiroz SA et al Mate selection provides similar genetic progress and average inbreeding than optimum contribution selection in the long-term. Aquaculture 2020;526:735376.

